# HTLV-1 Tax-induced NF-κB activation is negatively regulated by Ubiquitin-specific peptidase 20 (USP20)

**DOI:** 10.1186/1742-4690-8-S1-A129

**Published:** 2011-06-06

**Authors:** Junichiro Yasunaga, Frank C Lin, Xiongbin Lu, Kuan-Teh Jeang

**Affiliations:** 1 Molecular Virology Section, Laboratory of Molecular Microbiology, NIAID, NIH, Bethesda, Maryland, 20892, USA; 2 University of Texas MD Anderson Cancer Center, Houston, TX, 77030, USA

## 

Human T cell leukemia virus type 1 (HTLV-1) causes a fatal hematopoietic malignancy, adult T cell leukemia (ATL), and a viral oncoprotein Tax is considered to play the important roles in leukemogenesis through its potent activation of NF-κB. Protein ubiquitination is crucial for the proper regulation of NF-κB pathway. It is also known that the function of Tax is modified by ubiquitination, indicating that ubiquitination machineries contribute to the oncogenic mechanisms of ATL. We report that two ubiquitin-specific peptidases, USP20 and USP33, deubiquitinate TRAF6 and suppress IL-1β-induced NF-κB activation. We find that USP20 deubiquitinates Tax and inhibits Tax-induced NF-κB activation, consistent with Tax being a substrate of USP20. In HTLV-1-transformed cells, the transcription of USP20 is reduced compared with HTLV-1-negative T cells, and ectopic USP20 expression was found to inhibit the proliferation of an HTLV-1-transformed cell line, MT4. Our findings suggest USP20 is a key negative regulator of NF-κB signaling and can influence HTLV-1-induced leukemogenesis.

